# Mesenchymal Stem Cells From Mouse Hair Follicles Reduce Hypertrophic Scarring in a Murine Wound Healing Model

**DOI:** 10.1007/s12015-021-10288-7

**Published:** 2022-01-26

**Authors:** Hanluo Li, Mirjana Ziemer, Ivana Stojanovic, Tamara Saksida, Danijela Maksimovic-Ivanic, Sanja Mijatovic, Goran Djmura, Dragica Gajic, Ivan Koprivica, Tamara Krajnovic, Dijana Draca, Jan-Christoph Simon, Bernd Lethaus, Vuk Savkovic

**Affiliations:** 1 National “111” Center for Cellular Regulation and Molecular Pharmaceutics, Hubei Provincial Key Laboratory of Industrial Microbiology, Sino-German Biomedical Center, Hubei University of Technology, Wuhan, 430068 Hubei Province China; 2 Department of Cranial Maxillofacial Plastic Surgery, University Clinic Leipzig, 04103 Leipzig, Germany; 3 Clinic for Dermatology, Venereology and Allergology, University Hospital Leipzig, 04103 Leipzig, Germany; 4 Institute for Biological Research “Sinisa Stankovic” (IBISS) – National Institute of Republic of Serbia, University of Belgrade, Belgrade, Serbia

**Keywords:** Mesenchymal stem cells, Hair follicles, Outer root sheath, Syngeneic immunocompetent model, Wound healing, Murine wound model, Hypertrophic scarring

## Abstract

Wound healing of acute full-thickness injuries and chronic non-healing ulcers leads to delayed wound closure, prolonged recovery period and hypertrophic scarring, generating a demand for an autologous cell therapy and a relevant pre-clinical research models for wound healing. In this study, an immunocompetent model for wound healing was employed using a syngeneic murine cell line of mesenchymal stem cells cultured from the mouse whisker hair follicle outer root sheath (named moMSCORS). moMSCORS were isolated using an air-liquid interface method, expanded *in vitro* and characterized according to the MSC definition criteria - cell viability, *in vitro* proliferation, MSC phenotype and multi-lineage differentiations. Moreover, upon applying moMSCORS in an *in vivo* full-thickness wound model in the syngeneic C57BL/6 mice, the treated wounds displayed different morphology to that of the untreated wound beds. Quantitative evaluation of angiogenesis, granulation and wound closure involving clinical scoring and software-based quantification indicated a lower degree of inflammation in the treated wounds. Histological staining of treated wounds by the means of H&E, Alcian Blue, PicroSirius Red and αSMA immune labelling showed lower cellularity, less collagen filaments as well as thinner dermal and epidermal layers compared with the untreated wounds, indicating a general reduction of hypertrophic scars. The decreased inflammation, accelerated wound closure and non-hypertrophic scarring, which were facilitated by moMSCORS, hereby address a common problem of hypertrophic scars and non-physiological tissue properties upon wound closure, and additionally offer an *in vivo* model for the autologous cell-based wound healing.

**Graphic Abstract:**

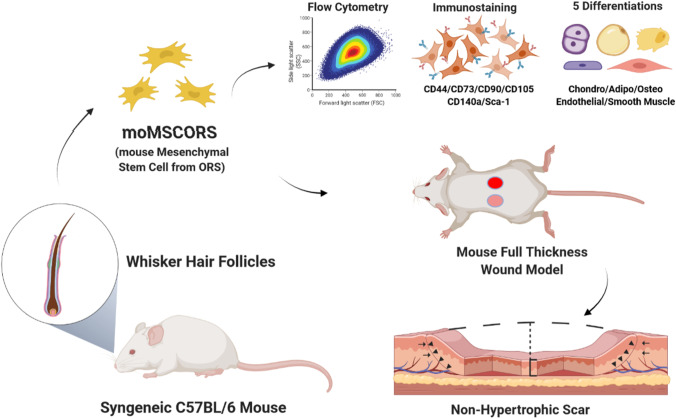

## Introduction

Skin and its appendages are continuously renewed and maintained, upkeeping the integrity and functions of protection, sensation, excretion, immunogenicity and synthesis, which are essential for homeostasis of the organism [1, 2]. Skin injuries are followed by complex and tightly orchestrated reactions of all cellular and extracellular components in a process known as wound healing [3]. Normal skin damage can be spontaneously healed by a regenerative process of 4 phases – hemostasis, inflammation, proliferation and maturation. The inflammatory phase is mediated by cytokines and pro-inflammatory cells, such as neutrophils, monocytes and macrophages, sequentially activating signaling cascades associated with angiogenesis, thrombosis, dermal ECM production and re-epithelialization [4, 5].

Injuries affecting deep dermis may lead to a delayed wound closure, prolonged recovery period and hypertrophic scarring due to the compromised regenerative elements necessary for the normal epithelialization and vascularization. Accordingly, extensive full-thickness injuries require clinical treatment such as surgical suturing and skin grafting in order to close the wound and prevent an infection and fluid loss [6]. Moreover, ulcers caused by chronic diseases can be treated with skin substitute against resistant ulcer progression and defective skin function [7]. If the healing process is not accomplished, the wound is likely to remain in a perpetual inflammatory state and develops into a non-healing wound, which is frequently associated with other chronic disorders and creates an enormous health burden worldwide [6–8].

The usual procedure for treating non-healing wounds is an autologous split-thickness skin autograft. The basic downside of this method is the invasive nature of harvesting by split-thickness biopsy, which causes a scar at the harvesting site and potential subsequent scarring of the applied area too [8, 9]. In search for less invasive treatments, tissue-engineered skin equivalents are produced using cultured autologous cell sources, including keratinocytes [10, 11]. These procedures involved expanded keratinocyte populations to treat large surface of extensive life-threatening burns [10]. Treatments using stem cells isolated from skin biopsy introduced a major turn in treatments of acute and large-size lesions altogether [12]. Those studies also indicated that the regenerative capacity of the skin largely relied not only on keratinocytes and melanocytes located in the basal layer of epidermis or on dermal fibroblasts, but also on the resident stem cells and precursors in different compartments of skin and its appendages [13]. In case of skin injury, those cells are being mobilized by the signals released from the sites of injury and they engage in wound healing modulation, tissue replacement as well as regeneration [6]. *In vivo* preclinical models of wound healing in murine remain widely used for studying complex biological processes and cellular interactions during the healing process [14]. In the course of proceeding towards clinical applications, regulatory requirements for cell-based therapies include pre-clinical testing in animal models in order to verify the *in vivo* safety and efficiency [15]. Same set of requirements, issued by the Committee for Human Medicinal Products (CHMP) in EMA, and certified by the Committee for Advanced Therapies (CAT). Typical *in vivo* safety and efficacy tests include xenogeneic transplantation of human cells to an immunocompromised animal.

Thus, along with the use of the state-of-the-art *ex vivo* methods for cell isolation and the development of *in vitro* cell engineering methods, mesenchymal stem cells (MSCs) have become endorsed as a supporting cellular component in wound healing therapies [16]. As an autologous cell source, MSC have shown capacity to set forth paracrine and trophic effects during the wound healing and repair process as well [5, 16, 17]. In the course of the inflammatory phase, a number of immune cells are involved, which are sequentially important for fibroblast/myofibroblast differentiation in the proliferative phase. MSCs appear to take part in moderating these processes [18]. Lately, a particularly notable role of MSCs in wound healing has been recognized, primarily due to their anti-inflammatory and trophic effects, which included developmental cues for other effects including angiogenesis and hematopoietic stem cell engraftment [5, 16, 17, 19, 20].

In a mouse wound healing model, MSCs were found to migrate systemically *in vivo* [21], and aggregate at the sites of lesion and inflammation [22]. Studies showed that the trophic and paracrine effect of MSCs in the animal model provided beneficial and therapeutic potential in terms of promoting topical regeneration of angiogenesis [19], hematopoietic stem cells engraftment [20] as well as exerting an anti-inflammatory [23], and immunosuppressive effect [24]. Those fine-tuning effects expanded the understanding of the MSC’s regenerative impact beyond that of the mere precursors for tissue replacement, which used to be the mainstream perception of the MSC function in injuries.

The very first echelon of MSCs that could be mobilized as a reaction to a skin injury are MSCs from the outer root sheath (ORS) of hair follicles, which are able to migrate towards the wound bed and participate in regeneration [25]. Moreover, hair follicle has gained a name “bone marrow of the skin” since it harbored putative heterogeneous stem cell populations such as Nestin^+^/Lgr6^+^ cells and MSCs [26–29], in human as well as in most mammals, including mouse whisker hair follicles [28–31]. Not less importantly, hair follicle MSCs are obtained non-invasively by hair plucking requiring minimal sampling amounts; also, they are available within a broad time window and expandable into high numbers, all adherent to patient-friendly approaches of personalized medicine. Recently, we have reported a robust procedure for culturing such MSCs from hair follicle ORS, given an abbreviation MSCORS. They displayed prominent *in vitro* negative modulation of monocyte differentiation towards anti-inflammatory macrophages, next to their scale-up potential and a multi-lineage differentiation capacity. All of this makes them a very competitive candidate for cell therapy and regenerative medicine [32, 33] .

This study deals with translating MSCORS into a mouse model as a part of a cell-based therapy for wound healing. To achieve this, we standardized an *ex vivo/in vitro* method to isolate MSC from the whisker hair follicle ORS of the immunocompetent C57BL/6 mice using the air-liquid outgrowth method for culturing MSCORS, named moMSCORS (mouse MSCORS). Further, we assessed their safety in a tumorigenicity study and their effect on wound healing based on trophic and immunomodulatory effects in a full-thickness-punch wound model. The *in vivo* studies were performed in a syngeneic context, comparable to autologous grafting. This set a base for the main aim of establishing a reliable mouse model for moMSCORS application *in vivo*, demonstrating their safety and an effect in wound healing as a part of the initial steps of their pre-clinical development.

## Methods

This study was approved institutionally by the Ethical Committee of Medical Faculty, University of Leipzig, DE (427/16-ek). Syngeneic mouse moMSCORS were isolated from the C57BL/6 mouse strain, to study the ORS-derived MSCs in immunocompetent syngeneic mouse system of the same strain, which is analog to autologous grafting. The C57BL/6 mice were obtained from the Core Unit for Animal Research of the Saxon Incubator for Clinical Translation (SIKT), Leipzig University. Young C57BL/6 mice (6-8 weeks old) (n=5) were sacrificed for other experimental purposes; the remaining skin parts were set at our disposal and they were used to isolate the follicles.

The *in vivo* experiments using C57BL/6 mice addressing the moMSCORS safety and their effect on wound healing were performed at the facility of the Institute for Biological Research “Siniša Stanković” (IBISS), University of Belgrade, Serbia. Animals were kept in the standard breeding conditions (non-specific pathogen free) with free access to food and water. The handling of animals and the study protocols were in agreement with the rules of the European Union and were approved by the University Animal Care and Use Committee at the Institute for Biological Research “Siniša Stanković”, University of Belgrade, Serbia (06-11/18).

### Isolation of mouse MSCORS (moMSCORS)

The rodent whisker hairs are located on whisker pads, the bilateral facial regions above the nose and the upper lip. Due to the anatomic bonds of mouse hair follicles with their dermal milieu, one cannot obtain them intact by plucking; the extraction typically results in a drastic depletion of the ORS. Thus, in this study we isolated hair follicle MSCs by dissecting an enzymatically pre-digested mouse facial skin, excising intact follicles and collecting them for purposes of moMSCORS isolation.

The facial skin area was disinfected by distilled water, 70 % ethanol, and PBS. The lip pad containing whisker hairs was harvested using dissecting scissors by incising from the point of the angulus or around the whisker pads. After a rough rinse with PBS, the attached muscle, subdermal fat and blood vessels were cautiously removed by scraping or excising until the whisker pad was stripped of the adjacent tissue. Each clean whisker pad was transferred to a new 35-mm petri dish containing 5 mL of 2 mg/mL Collagenase V, which as a pan-Collagenase partially digests multiple collagen types (Sigma-Aldrich Chemie GmbH, Steinheim, DE), and incubated at 37 °C for 3 h.

After Collagenase digestion, the whisker pad tissue was loosened and swelled, with visible robust whisker hair follicles and a visible large follicle–sinus complex. In order to obtain the hair follicle ORS, the dermal cavernous sinus was removed. Hair shaft was clamped by forceps to stabilize the follicle–sinus complex and the lower 3/4 of the cavernous sinus was superficially incised with particular care not to damage the ORS of the follicle. The whisker hair shaft was clipped by a pair of forceps from the outer side and pushed in the proximal direction (opposite of hair growth). Once the whole hair follicle and a short part of the hair shaft became exposed at the inner dermal side of the skin, the shaft was clipped close to the dermal side and further pulled in proximal direction to extract a follicle with an intact ORS.

Isolated whisker hair follicles were placed in the new 35-mm petri dish containing DMEM with Penicillin/Streptomycin (Pen/Strep) (100 U/mL/100 µg/mL). The base part with dermal papilla was cut off with a surgical scalpel.

The mouse hair follicles were processed using the same procedure as previously described [32]. Briefly, 10 whisker hair follicles were seeded on to Corning Transwell membrane (Corning Inc., New York, NY, USA) of 6-well format, which was filled with 0.9 mL MSCORS Expansion Medium (DMEM low glucose + 10 % fetal bovine serum (FBS) + 2 mM L-glutamine + Pen/Strep (100U/mL/100µg/mL)) to set up an air-liquid-interface culture. Hair follicles were incubated under hypoxic conditions (5 % O_2_, 5 % CO_2_) at 37 °C for 2 days. The medium was replaced with 1.2 mL of fresh medium. After 7 days, the migration and further outgrowth of the moMSCORS onto the Transwell membrane was visible, followed by forming a cell monolayer. After 17-24 days, at 50-80 % confluence, the cells were detached using 0.04 %/0.03 % Trypsin/EDTA (PromoCell GmbH, Heidelberg, DE), and subcultured in 6-well plates. The moMSCORS required 2-3 days to recover from the trypsinization. After the recovery period, moMSCORS proliferated rapidly and they were continuously subcultured for at least 10 passages. In this study, moMSCORS before P6 were used for characterization and differentiation tests.

### Cultivation and Proliferative Characterization of moMSCORS

moMSCORS were cultivated on T75 culture flask and continuously subcultured at 90 % confluence for 6 passages. Cell counts were obtained using a cell counter (CASY Cell Analyzer, OMNI Life Science GmbH, Bremen, DE), herein indicating the cell yield in each passage. Cell viability in terms of mitochondrial dehydrogenase activity was assessed using tetrazolium-based salt WST-1 (4-[3-(4-Iodophenyl)-2-(4-nitro-phenyl)-2 H-5-tetrazolio]-1, 3-benzene disulfonate) assay as previously described [32]. Positive correlation of the converted WST-1 signal intensity, cell viability and cell number has previously been shown in the same study. Briefly, moMSCORS were split and seeded in a flat bottom 96-well plate with a density of 5000 cells per 1 well (1.56 × 10^4^ cells/cm^2^). After attachment, the cells were incubated with 10 % WST-1 for 60 min at 37 °C. The absorbance of the supernatant was measured using a spectrophotometry plate reader at the wavelength of 450 nm with 620 nm and against a blank background control.

### Surface Marker Analysis Using Fluorescence-Activated Cell Sorting (FACS) and Immunofluorescent Staining

To determine the phenotype of moMSCORS, FACS analysis was employed and moMSCORS in passage lower than 3 were labelled with fluorochrome-conjugated rat-anti-mouse antibodies for CD44, CD73, CD90, CD105, Sca-1 (Stem Cell Antigen-1), CD140a, CD117, CD45, and CD31. Labeled cells were re-suspended and fluorescence intensity was analyzed by the means of flow cytometer BD FACS Canto II and FlowJo 10.0 software (BD Biosciences, San Jose, CA, USA).

For purposes of immunofluorescent staining, moMSCORS were seeded onto the 8-well chamber slides (ThermoFisher Inc., Waltham, MA, USA), fixed with 4 % paraformaldehyde (PFA), and blocked with 10 % normal goat serum (ThermoFisher Inc., Waltham, MA, USA). The cells were labelled with 1:100 diluted fluorochrome-conjugated or biotin-conjugated anti-mouse antibody for CD44, CD73, CD90, CD105, Sca-1, nestin, CD117, CD133 and CD45 (Table 1). After washing, cells stained with biotin-conjugated antibodies were incubated with streptavidin-PE (BD Biosciences, San Jose, CA, USA) and counterstained with 4′, 6-diamidino-2-phenylindole (DAPI), ThermoFisher Inc., Waltham, MA, USA). After mounting, the cells were imaged using a Keyence BZ-9000 Fluorescence Microscope.

**Table 1:** Primary antibodies used in this study

Antibody	Immunoglobulin, Clone	Fluorochrome	Manufacturer
Anti-Mouse CD44	rat IgG2bκ, Clone IM7	APC-eFluor 780	ThermoFisher Scientific Inc., Waltham, USA
Anti-Mouse CD44	rat IgG2bκ, Clone IM7.8.1	Biotin	ThermoFisher Scientific Inc., Waltham, USA
Anti-Mouse CD73	rat IgG1, Clone TY/11.8	eFluro 710	ThermoFisher Scientific Inc., Waltham, USA
Anti-Mo/Rat CD90.1	mIgG2aκ, Clone HIS51	PE-Cy5	ThermoFisher Scientific Inc., Waltham, USA
Anti-Mouse CD105	rat IgG2aκ, Clone MJ7/18	Biotin	BioLegend Inc., San Diego, USA
Anti-Mouse CD105	rat IgG2aκ, Clone MJ7/18	PE/Cy7	BioLegend Inc., San Diego, USA
Anti-Mouse Sca-1	rat IgG2aκ, Clone D7	APC	Miltenyi Biotec GmbH, Bergisch Gladbach, DE
Anti-Mouse Sca-1	rat IgG2aκ, Clone E13-161.7	Biotin	BioLegend Inc., San Diego, USA
Anti-Mouse CD45	rat IgG2b, Clone 30-F11	Biotin	Miltenyi Biotec GmbH, Bergisch Gladbach, DE
Anti-Mouse CD45	rat IgG2bκ, Clone 30-F11	PE-Cy7	ThermoFisher Scientific Inc., Waltham, USA
Anti-Mouse CD31	rat IgG2aκ, Clone 390	APC	ThermoFisher Scientific Inc., Waltham, USA
Anti-Mouse CD117	rat IgG2aκ, Clone 2B8	PE-Cy7	ThermoFisher Scientific Inc., Waltham, USA
Anti-Mouse CD117	rat IgG2Bκ, Clone 2B8	Biotin	ThermoFisher Scientific Inc., Waltham, USA
Anti-Mouse CD133	rat IgG1κ, Clone 13A4	Biotin	ThermoFisher Scientific Inc., Waltham, USA
Anti-mouse CD140a	Rat / IgG2aκ, Clone APA5	APC	ThermoFisher Scientific Inc., Waltham, USA

### moMSCORS Differentiation Assays

The moMSCORS were cultivated and expanded until passage 4, to be subsequently induced towards multi-lineage differentiations.

#### Adipogenic Differentiation

DMEM (1 g/L glucose) was used with 10 % FBS, 1 µM dexamethasone, 500 µM IBMX, 100 µM indomethacin, 10 µM/mL insulin, 1 % non-essential amino acids and 1 % L-glutamine. moMSCORS were seeded in the culture plate at 6 × 10^3^/cm^2^ density and differentiated for 14 and 21 days in normoxic conditions (5 % CO_2_). The cells differentiated towards adipose lineage were stained with Oil Red O dye to detect the fatty acid vesicles.

#### Chondrogenic Differentiation

moMSCORS were differentiated in pellet culture, and 2.5 × 10^5^ cells were centrifuged for 10 min. The cells were cultured in hypoxic conditions (5 % CO_2_, 5 % O_2_) for 21 and 28 days. The cartilage differentiation medium was based on DMEM/F12 medium, supplemented by 1 % FBS, 10 ng/mL TGF-β1, 10 ng/mL bone morphogenetic protein BMP-7, 10^−7^ M dexamethasone, 50 ug/mL ascorbic acid (ASC), 50 ug/mL Na-pyruvate, 1 % non-essential amino acids, 1 % L-glutamine. Upon differentiation towards chondrogenic lineage, the pellets were histologically sectioned and stained by Alcian Blue (Sigma-Aldrich Chemie GmbH, Steinheim, DE) to detect the formation of proteoglycans.

#### Osteogenic Differentiation

DMEM (1 g/L glucose) was used as the matrix in osteoblast differentiation medium, with 10 % FBS, 2 × 10^−7^ M dexamethasone, 50 ug/mL ASC, 10 mM β-glycerophosphate (β-Gly) and 1 % L-glutamine. moMSCORS were seeded onto a culture plate at density of 2 × 10^4^/cm^2^, and differentiated for periods of 21 and 28 days in hypoxic environment (5 % CO_2_, 5 % O_2_). The extracellular calcium phosphate deposition was detected by Alizarin red, the activity of Alkaline Phosphatase (ALP) was detected by BCIP/NBT, and the calcium phosphate deposition was detected by the cresolphthalein complexone (CPC) assay (Greiner Diagnostic GmbH, Bahlingen, DE).

#### Endothelial Differentiation

DMEM (1 g/L glucose) was used as matrix in endothelial cell differentiation medium supplemented by 10 % FBS, 0.5mM 2-mercaptoethanol, 30 ng/mL VEGF, 5 ng/mL bone morphogenetic protein BMP-4, 1 % L-glutamine. moMSCORS were seeded in the culture plate at a density of 2.5 × 10^4^/cm^2^, and differentiated for 21 and 28 days in hypoxic environment (5 % CO_2_, 5 % O_2_). CD31 immunofluorescence staining was used to detect endothelial function in differentiated cells.

#### Smooth Muscle Differentiation

DMEM (1 g/L glucose) was used as matrix in the smooth muscle cell differentiation medium, with 10 % FBS, 10 ng/mL TGF-β1, 1 % L-glutamine. moMSCORS were seeded in a culture plate at density of 2.5 × 10^4^/cm^2^, and differentiated for 21 and 28 days in hypoxic environment (5 % CO_2_, 5 % O_2_). The cells differentiated to a smooth muscle lineage were stained by the alpha smooth muscle actin (αSMA) antibody.

### moMSCORS Grafting for Purposes of Wound Healing

To study the contribution of moMSCORS to wound healing in the syngeneic mouse model, moMSCORS grafting procedure onto the full-thickness-punch wounds was used. C57BL/6 mice at the age of 8-10 weeks were divided into two groups:

- Control Group: wounds treated with PBS (20 animals)
- Treated Group: wounds treated with moMSCORS (20 animals)

Full-thickness wounding procedure by a punch-biopsy was applied to the dorsal area of mice. Prior to the wounding, all animals were anesthetized using intraperitoneal injection of the mix of Ketamine (80 mg/kg) and Xylazine (10 mg/kg). Hairs from the dorsal skin were shaved using electric clippers, and disinfected using 70 % ethanol. The mouse was positioned laterally, and the dorsal skin was folded up in the middle and laid at the bottom of a 9-cm petri dish. Using a 5mm diameter punch biopsy tool, a full-thickness wound was induced by punching through both sides of the fold of the dorsal skin, hereby inflicting two identical bilateral wounds of the same size.

moMSCORS were cultivated and harvested by trypsinization, and re-suspended in PBS in density of 2 × 10^7^ cells/ml. 10^6^ cells with 50 uL PBS were carefully applied to each mouse from the treated group, evenly distributed across the wound surface, and the animals in both groups were laid flat for 30-60 min to avoid liquid leakage until they came awake. The wound morphology and size were documented with a digital camera on day 1, 3, 5, 7, 9, 11, 14 subsequent to cell grafting. During observation, the mice were anesthetized and placed flat with wound area and camera on tripod in fixed positions, using identical light source throughout the photo-documentation. The wound was exposed to the camera maintaining the same distance, aperture, exposure and a ruler adjacent to the wound as scale bar. Both groups were sacrificed on day 14, the wound skin was excised and embedded in paraffin for purposes of histological analysis.

### Analyzing the Wounds via ImageJ and Scoring

With standardized documentation photos across the time points, the sizes of the healing wounds in both groups were analyzed using ImageJ analysis software (Version 1.53a, https://imagej.nih.gov/ij/). Briefly, the scale was set according to the ruler placed next to the wound in each photo. The area was outlined by following the clear edge of the wound. A closed wound, or a wound with a margin area covered with thick fibrin scab, exhibited an unrecognizable shape and they were therefore excluded from the evaluation. The surface of the clear area of non-closed wounds was assessed by the means of the ImageJ software.

The wound conditions, in terms of inflammation, granulation, angiogenesis, scab thickness, and wound closure, were reviewed and scored by a senior clinical dermatologist at Clinic and Polyclinic for Dermatology, Venereology and Allergology, University Hospital Leipzig, based on clinical experience and an Innovative Wound Score system described by Strauss et al. in 2016 [34]. The scoring criteria are displayed in Table 2.

**Table 2:** Clinical scoring criteria applied for evaluation of the mouse punch wound

Inflammation (4 Quarters)	Granulation (4 Quarters)	Angiogenesis (4 Quarters)	Wound Closure
Pale=0	No=0	No=0	No=0
Red=1	Yes=1	Yes=1	Yes=1
Not applicable=N	Not applicable=N	Not applicable=N	

### Analyzing the Wounds via Histological Staining

Histological staining was used to study the healed wounds. For this purpose, the samples of random 5 individuals from each group were histologically analyzed. Briefly, on day 14, mouse skin tissue containing wound bed was harvested, fixed overnight in 4 % PFA, embedded in paraffin blocks and cut into 5 μm-thick sections. After de-paraffinization and rehydration, the sections were stained with Hematoxylin and Eosin (H&E, Carl Roth GmbH, Karlsruhe, DE), Alcian Blue (Sigma-Aldrich GmbH, Schnelldorf, DE), and PicroSirius Red. For purposes of PicroSirius Red Staining, mouse skin section was stained with Weigert’s Haematoxylin Working Solution (Carl Roth GmbH, Karlsruhe, DE) and 1 % PicroSirius Red (Carl Roth GmbH, Karlsruhe, DE). After washing and dehydration, the stained section was cleaned with xylene and mounted in DPX Mounting Solution (Carl Roth GmbH, Karlsruhe, DE). For immunostaining of αSMA, the deparaffinized and rehydrated sections were blocked in 10 % Normal Goat Serum (ThermoFisher Scientific Inc., Waltham, USA), and incubated with rabbit-anti-mouse αSMA antibody (Abcam Plc, Cambridge, USA; 1:100 dilution) overnight. After washing, the sections were incubated with 2nd antibody of AlexaFluor® 594-conjugated goat-anti-rabbit IgG (ThermoFisher Inc., Waltham, MA, USA; 1.0 mg/mL, 1:200 dilution) and 4′, 6-diamidino-2-phenylindole (DAPI; ThermoFisher Inc., Waltham, MA, USA; 1:400 dilution). The stained and immunofluorescently labelled histological sections were imaged using Keyence BZ-9000 Fluorescence Microscope (Keyence GmbH, Neu-Isenburg, DE).

### Safety Study *In Vivo*

In order to pilot-test the tumorigenic risk of the moMSCORS application, syngeneic cell line from C57BL/6 mice was inoculated to the four-week old C57BL/6 animals, hereby presenting the set-up analogue to an autologous application. Total of 39 animals were involved in the safety study, structured into three treatment groups inoculated with moMSCORS (n=8, n=8, n=13), one positive control group (n=5) inoculated with B16-F10 mouse melanoma tumor cell line and one sham group (n=5).

Injections of moMSCORS suspension, each containing 10^6^ cells, were applied to animals intraperitoneally and subcutaneously, alone and combined. The animals were kept in standard animal facility conditions with food and water *ad libitum*. They were examined for habitus and behavior, weighed and photo-documented on weekly basis. After 26 weeks, the animals were sacrificed by the means of cervical dislocation, examined and dissected. All organs and the peritoneal membrane were assessed visually and palpatorily by a team composed of a resident veterinarian and five biologists. All observations were carried out based on consensus of all team members. All isolates were photo-documented and archived.

### Statistical Analysis

Results were analyzed statistically using an unpaired t-test or non-parametric Mann-Whitney test. Normal distribution and homogeneity of variance of datasets were verified using a Shapiro-Wilk normality test. p values lower than 0.05 were considered statistically significant (*p<0.05, **p<0.01, ***p<0.005).

## Results

### moMSCORS Isolation and Characterization

**Fig. 1:**
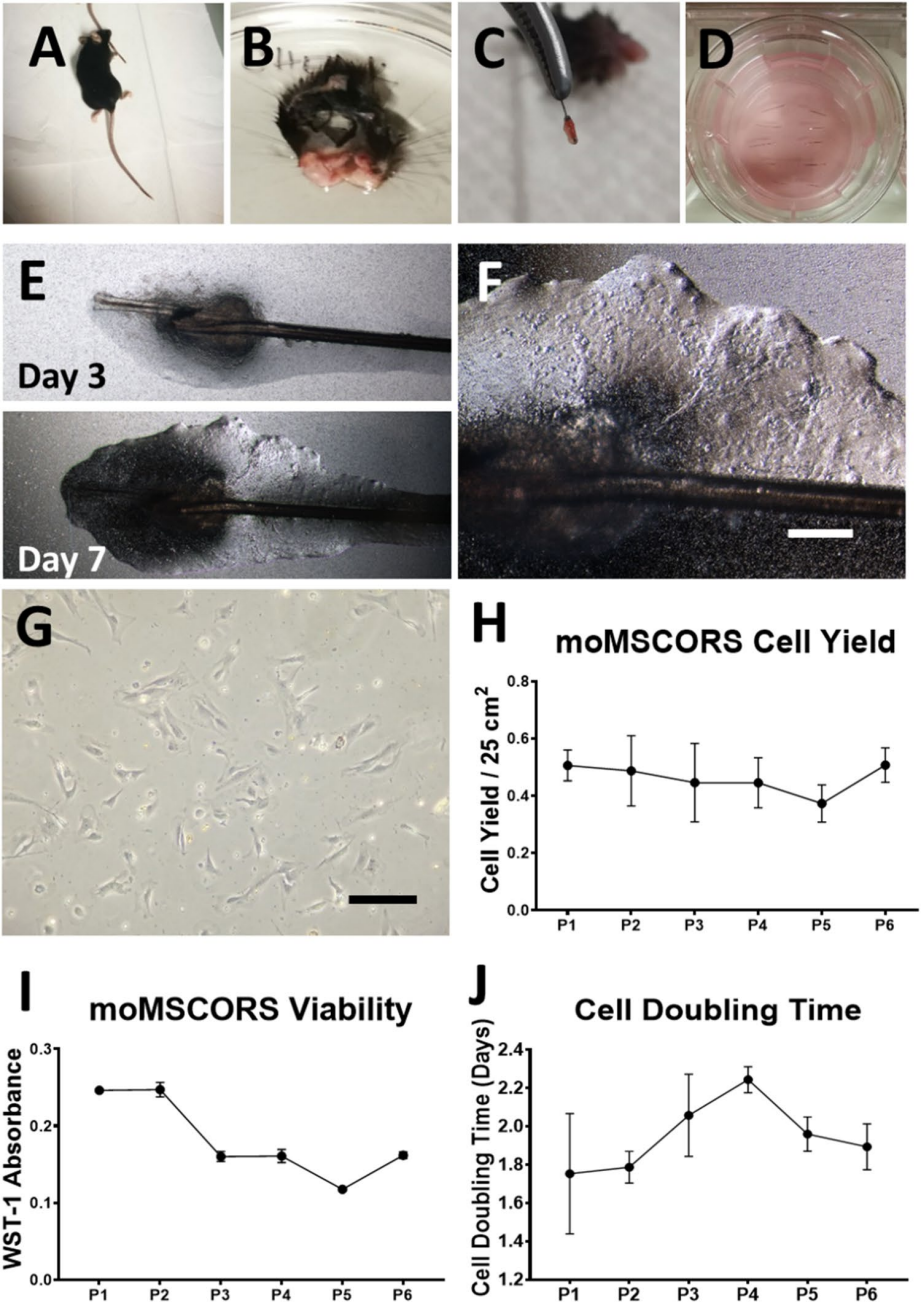
Isolating and cultivating syngeneic mouse MSCORS (moMSCORS) from C57BL/6 mice. (**A**) Young C57BL/6 Mice (6-8 weeks old, n=5) were sacrificed, and the whole facial skin was disinfected. (**B**) The upper angulus oris containing the lip pad was excised. (**C**) After collagenase digestion, the whisker pad tissue was loosened and swelled, and the whole follicle–sinus complex was plucked from the skin. (**D**) Under the dissection microscope, the hair follicle ORS was isolated out of the follicle–sinus complex by incising open the follicle sinus capsule, and seeded onto the porous Transwell membrane. (E) ORS cells migrated out and formed a monolayer onto the porous membrane on Day 3 and Day 7. (**F**) Larger magnification of cell monolayer. (**G**) After confluence and harvesting, the moMSCORS attached onto the culture flask and proliferated as primary cell lines (p0). moMSCORS were rapidly expanded *in vitro* and subcultured for 6 passages upon confluence, with cell yield (**H**), cell mitochondrial activity (**I**) and cell doubling time (**J**) at passage 1-6. Data are shown as mean ± SD. Scale bar: (**F**,**G**) 100 μm

moMSCORS were isolated from the whisker hair of the C57BL/6 mouse, which was obtained from the complex of the hair follicle and the sinus from enzymatically digested facial skin, as shown in Fig. 1A-C. Typically, 20 hair follicles were obtained from two whisker pads of a single mouse. After 2 days, the heterogeneous ORS cells began to migrate out of the hair follicle and adhered to the Transwell membrane (Fig. 1D-F). Within 31.25±7.98 days, the outgrowth method used in this study yielded 0.48±0.31 million cells per one hair follicles in P0, as represented in Fig. 1G. After the established primary cultivation, moMSCORS showed rapid cell proliferation and viability. By using continuous passage and cell viability assay WST-1, moMSCORS yielded 2.18±0.69 × 10^4^ cells/cm^2^ in the course of 1-6 passages, with the corresponding cell mitochondrial activity and cell doubling time of 1.94±0.15 days, which are depicted in Fig. 1H-J.

### moMSCORS Surface Marker Expression

**Fig. 2:**
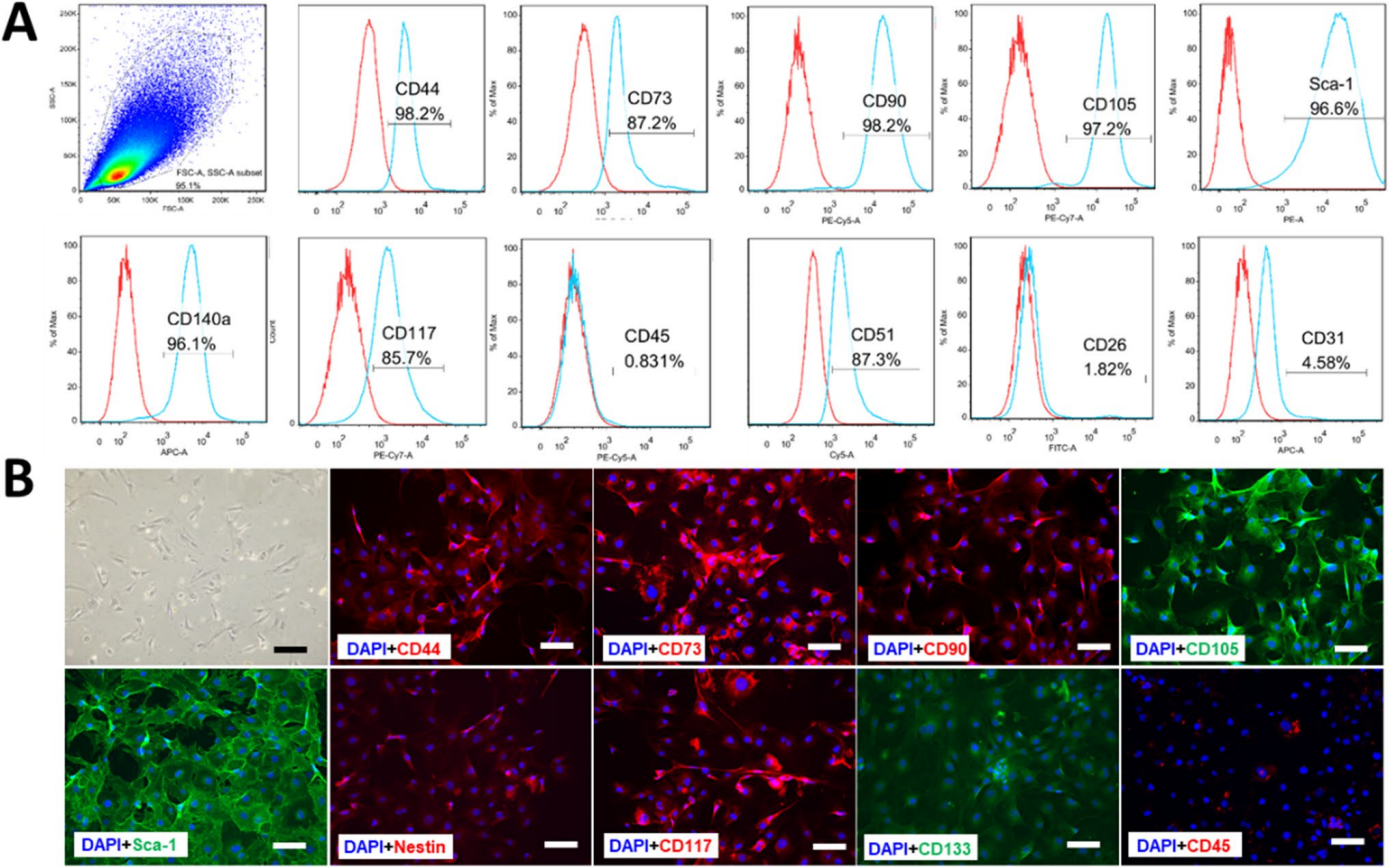
Surface biomarkers expression profile of moMSCORS. The phenotypic characterization of moMSCORS was investigated using flow cytometry and immunofluorescent staining to identify the surface marker expressions according to the ISCT MSC Criteria. (**A**) Representative plot graph and histograms of moMSCORS FACS analysis were exhibited. moMSCORS was stained positive with CD44, CD73, CD90, CD105, Sca-1, and CD140a, and was partially positive with CD117. Faint staining of CD31 and negative staining of CD45 was observed. (**B**) Using immunofluorescent staining moMSCORS expression of cell markers was visualized in terms of CD44, CD73, CD90, CD105, Sca-1, nestin, CD117, CD133, and negative for CD45. Scale bar corresponds to 50 μm, magnification 20x

After obtaining stable primary culture, the moMSCORS cell populations were defined and the phenotype was characterized. Flow cytometry using anti-mouse antibodies was employed to identify the moMSCORS surface marker expression. FACS results (Fig. 2) indicated that high portion of moMSCORS were expressing MSC biomarkers, including CD44 (70.4±19.80 %), CD73 (88.1±3.21 %), CD90 (97.1±1.42 %) and CD105 (93.9±2.33 %), in full accordance with the expression of MSC-relevant positive markers defined by the International Society for Cell Therapy (ISCT) [35]. moMSCORS also expressed Sca-1 (92.4±3.01 %) and CD140a (90.67±4.67 %) in high quota, as shown in Fig. 2A. Further biomarkers were also characterized in moMSCORS with intensive immunostainings of CD44, CD73, CD90, CD105, Sca-1, nestin, CD117, CD133, as well as the negative staining for CD45 (Fig. 2B).

### Five-lineage Differentiations of moMSCORS

**Fig. 3:**
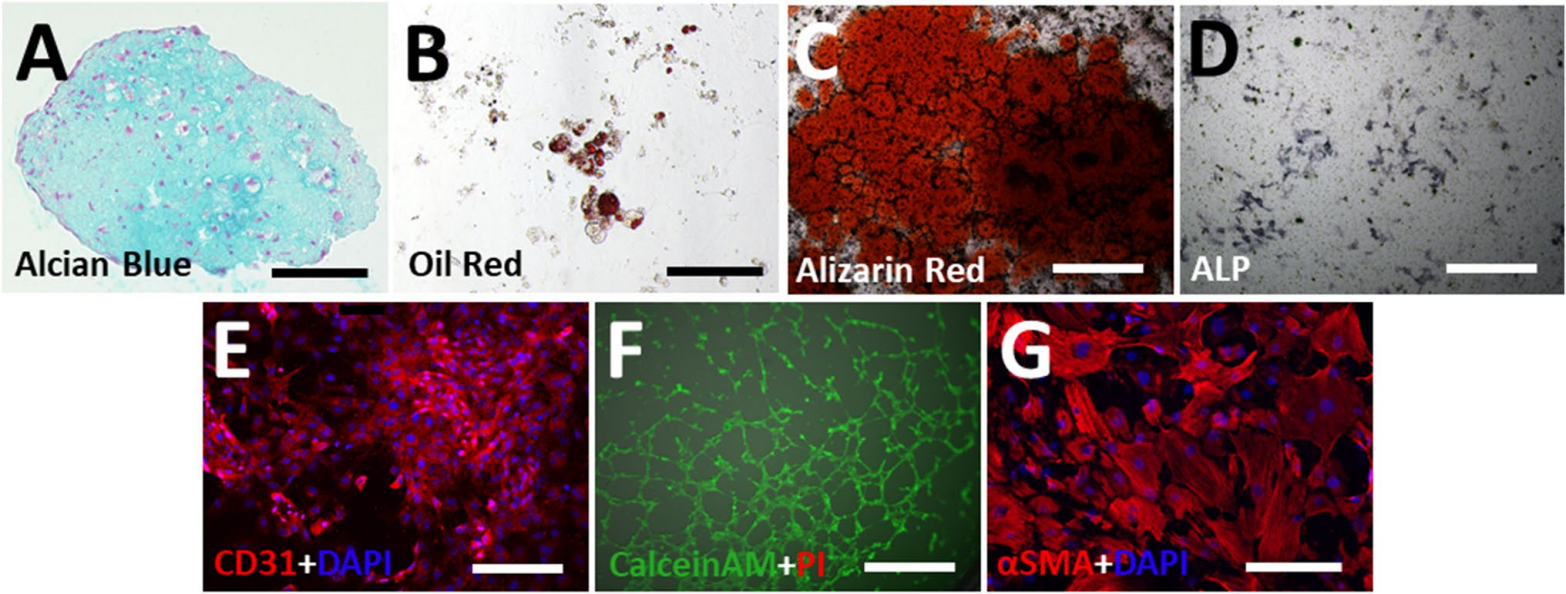
Differentiation Assays of moMSCORS. Differentiation capacities of moMSCORS. moMSCORS exhibited differentiation potentials towards 5 lineages including Chondrogenic, Adipogenic, Osteogenic, endothelial cells and smooth muscle cells. (**A**) Chondrogenic differentiation for 21 days using pellet culture, and the production of proteoglycan was detected using Alcian Blue staining. (**B**) Adipogenic differentiation for 21 days, the lipid vesicles were detected using Oil Red O staining. (**C,D**) Osteogenic differentiation of moMSCORS after 21 days induction. The calcium deposition was detected using Alizarin Red staining, and the activity of alkaline phosphatase visualized by the BCIP/NBT assay. (**E,F**) Endothelial differentiation of moMSCORS for 21 days was verified using CD31 immunostaining, and the vascular anastomosis was detected using the tube forming assay with Live/Dead assay (Calcein AM/Propodium Iodide). (**G**) Smooth muscle cell differentiation for 21 days, which was detected using αSMA immunostaining. Scale bar corresponds to (**A,B,G**) 100 μm, (**C,D,F**) 500 μm, (**E**) 200 μm; magnification (**A,B,G**) 20x, (**C,D,F**) 4x, (**E**) 10x

To confirm the moMSCORS phenotype, five-lineage differentiation assays including chondrogenic, adipogenic, osteogenic, endothelial and smooth muscle differentiations (Fig. 3) were used to investigate whether the moMSCORS exhibited key properties of the tri-lineage differentiations in accordance with the ISCT Criteria and the further differentiation potential that has been reported earlier in their human analogue MSCORS [32].

Using the same protocols as for human MSCORS [32], moMSCORS differentiated from a multipotent state into 5 mesenchymal lineages. Nonetheless, labelling intensity in histological and immunofluorescence assessment revealed that not all of the five lineage differentiations were as pronounced as in human MSCORS. Chondrogenic differentiation worked quite intensively. Although chondrogenic pellets formed by the moMSCORS were relatively small compared to those developed by human MSCORS, the cartilaginous ECM deposition in moMSCORS was more intensive and demonstrated a more clearly pronounced cartilage-like pattern (Fig. 3A). Interestingly, moMSCORS were nearly deficient in adipogenic differentiation, and only sporadically differentiated adipocytes stained by the Oil Red O were observed (Fig. 3B). Alizarin Red and ALP Assay showed a lower calcium production and less ALP-positive cells in moMSCORS after osteogenic differentiation (Fig. 3C, D). CD31 immunofluorescent staining and angiogenic assay showed an effective differentiation towards endothelial cell lineage (Fig. 3E, F). Smooth muscle differentiation was also shown by positive staining of αSMA (Fig. 3G).

In short, the syngenic mouse moMSCORS cell culture was isolated from C57BL/6 mice hair follicle using adapted MSCORS outgrowth isolation method. moMSCORS were characterized and defined as mouse MSC lineage that fits the ISCT-defined criteria for human MSCs: adherence, proliferation, marker expression and lineage differentiation. Herewith, mouse moMSCORS presented a cell source analogue to the human MSCORS and qualified for the next phase of *in vivo* experiments that would evaluate their safety and functionality in wound healing, each in a separate mouse model.

### moMSCORS-based Mouse Wound Healing Model: Wound Size

**Fig. 4:**
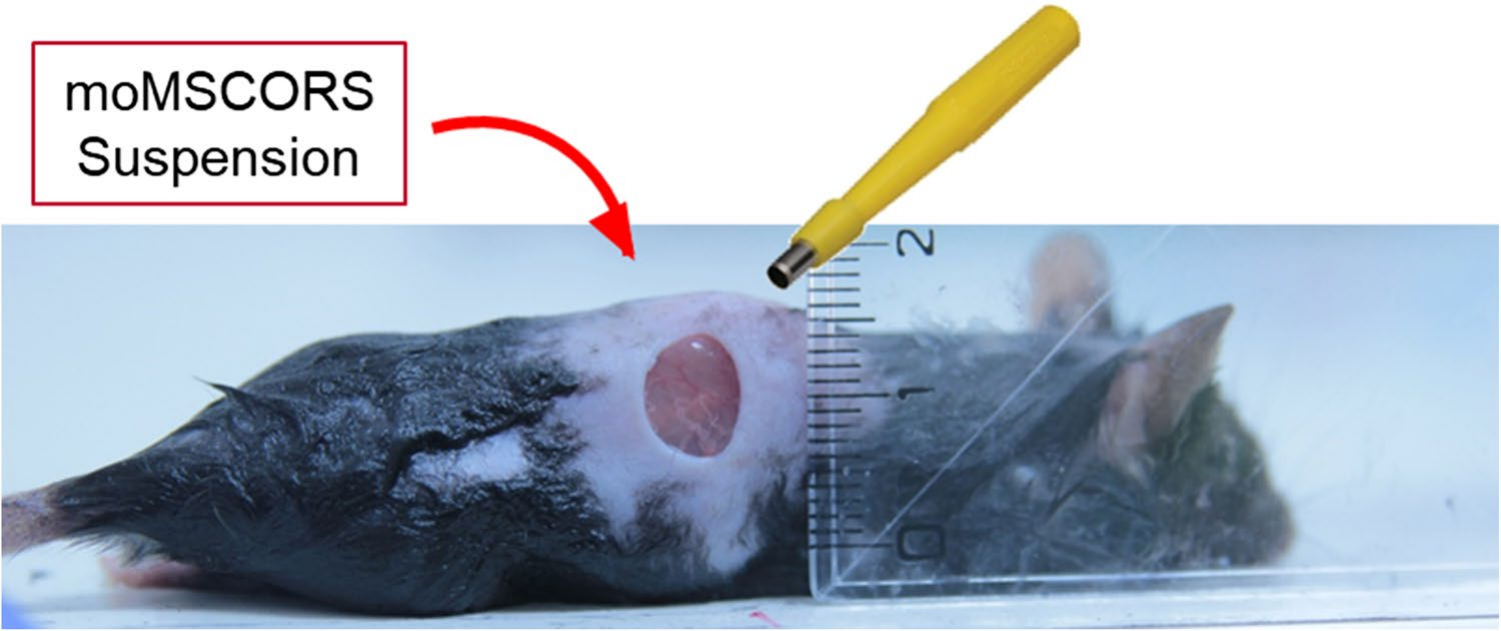
Demonstration of wound healing model using moMSCORS. moMSCORS (n=17) were applied on the induced full-thickness wound on the one side of the bilateral dorsum skin of C57BL/6 mice (n=27). Young healthy C57BL/6 mice (3 months) were chosen for this study. Using punch biopsy with the diameter of 5 mm, artificial wound was induced by punching through both layers of the dorsal skin. 10^6^ moMSCORS were resuspended with 50 uL PBS, and carefully applied onto the wound. PBS without cells was used in the untreated group. The mice were kept, and wound size was documented using camera on the day 1, 3, 5, 7, 9, 11, 14 after the cell seeding with a ruler aside for scaling

**Fig. 5:**
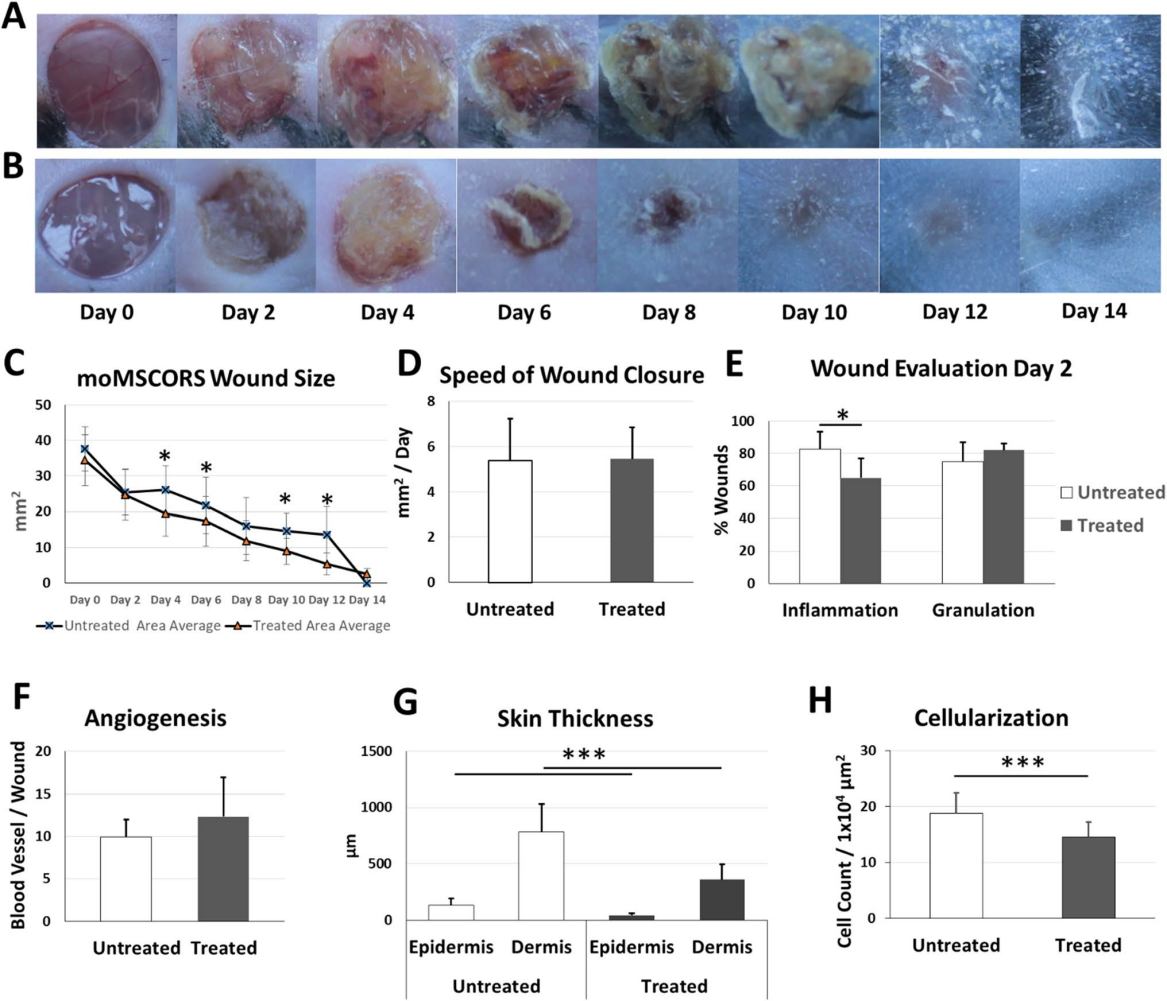
Time point analysis in the course of moMSCORS Wound Healing. Photo documentation of the wounds in the treated and untreated groups. Semi-quantitative measurement of wound size and wound closure using photo documents for analyzing the wound size, healing speed. Semi-quantitative analysis of the wounds based on the photos and clinical scoring evaluation in the first 2 days. Show case of mouse and wounds (**A**) without moMSCORS treatment and wounds (**B**) with moMSCORS treatment. (**C**) Wound size quantitatively measured over course of wound healing. (**D**) Calculated speed of wound healing using the slope of the linear regression of the wound closure curve. (**E**) Semi-quantitatively evaluation of wound bed were examined, and wound inflammation, granulation were presented. (**F**) Wound angiogenesis in terms of calculated average number of the visible new blood vessel on wound bed. (**G**) Quantitative measurement on the thickness of epidermis and dermis of untreated and treated groups. (**H**) Cellularization of the untreated and treated wound beds. Data are shown as mean ± SD (* p<0.05, ***p<0.001)

To study the effects of moMSCORS on the wound healing process, a mouse full-thickness wound model was used (Fig. 4). 10^6^ moMSCORS in PBS suspension were applied onto the wound on one side of the bilaterally punched dorsum skin wound of the C57BL/6 mice. The wound size was documented by a camera on day 1, 3, 5, 7, 9, 11, and 14 after the cell seeding. As shown in Fig. 5A, B, during 14 days of wound healing experiments with documentation it was clear that both the moMSCORS-treated and the untreated group underwent a typical wound healing process consisted of hemostatic, inflammatory, proliferative and regenerative maturation phases. However, the healing patterns e.g. wound closure speed differed between the two groups according to the visual inspection. The untreated group displayed larger and less contracted wound compared to that of the treated group. The crust in the untreated group formed from day 2 appeared thicker than in the treated group, with more intensive exudation and scabbing. The treated group showed a trend of displaying lower level of inflammation at the early stage of wound healing, as well as a more pronounced contraction and a thinner fibrin crust in the proliferative stage, compared to the untreated group. In exemplary cases, the scab discharge took place earlier in the treated group on day 8.

To investigate the wound changes over a 14-day monitoring period, the documented images were analyzed using ImageJ software in order to semi-quantitatively assess the wound size and wound closure (Fig. 5C). During day 4 to day 12, the wound size in the untreated group was 30–150 % larger than in the treated group. Wound size in the moMSCORS treated group was significantly smaller than in the untreated group, on day 4, day 6, day 10 and day 14 (by 25.82 % for day 4, ***p=0.0014; by 20.26 % for day 6, *p=0.0469; by 38.34 % for day 10, **p=0.007 ; by 60.63 % for day 12, *p=0.026). The treated group showed linear and mild wound size change over the 14-day inspection period. Rapid decrease of wound size occurred in the untreated group on day 2 and day 14. The decrease in wound size on day 2 was due to the stronger wound contraction on the day 2 post-wounding, and the decrease on day 14 owed to the regular process of scab exfoliation in the untreated group.

The pace of wound closure was defined as the slope of the changing curve of wound size in Fig. 5C. It showed comparable dynamics between the treated and untreated group with non-significant differences (p=0.24, Fig. 5D), notwithstanding the differences in wound size (Fig. 5D).

### Safety Study *In Vivo*

The experimental animals in the group inoculated with B16-F10 tumor cells developed observable and palpable solid tumors in both subcutaneously and intraperitoneally inoculated animals two to four weeks after inoculation. Peritoneum of the animals injected with B16-F10 group displayed solid tumors spread along the peritoneal membrane. In the control and moMSCORS-injected group, no signs of tumors were observed after 26 weeks upon inoculation (not shown). All experimental animals in the control group and group exposed to moMSCORS displayed a healthy habitus and normal activity. Tumors were neither visually nor palpatorily detectable in the living animals (not shown).

The weight of the sham-treated and moMSCORS-exposed group remained comparable at the same time points. Upon sacrificing and dissecting, the peritoneum showed an absence of tumors in the inner cavity of all animals. The isolated lung, heart, liver, pancreas, kidneys and bladder showed no signs of tumor presence according to the consensus of the investigating team (not shown).

### moMSCORS-based Mouse Wound Healing Model: Wound Evaluation

To investigate the clinical features of the wound during the monitoring period, the documented photographs of untreated and treated wounds on day 2 were evaluated by a dermatologist (Fig. 5E, H). moMSCORS-treated group showed significantly lower inflammation score compared to those of the untreated group, as well as differences in edema and cellularity.

The inflammation scoring of the wounds in the early healing process in the day 2 revealed that both the treated and the untreated groups developed visible inflammation of the wound area (Fig. 5A). Inflammation was observed in all analyzed wounds in both groups, with red-inflamed granulation tissue underneath the fibrin crusts. 82.5±10.89 % of untreated wounds displayed intensive inflammation of the wound bed, whereas fewer of the treated wounds, 65±11.73 % of showed intensive inflammation (*p=0.033). 75.07±0.60 % of untreated wounds and 81.94±4.16 % of treated wounds displayed a large, non-significantly different amount of *de novo* granulation tissue in the early period of wound healing (p=0.42, Fig. 5E).

All the wounds displayed active angiogenesis in the early phase of the wound healing. The neovascularization and angiogenesis were clearly detectible underneath the fibrin crusts of the wound (Fig. 5A, B). In untreated wounds, 9.91±2.06 *de novo* blood vessels were observed in average, comparable to 12.32±4.63 in the treated wound beds (p=0.22, Fig. 5F).

### moMSCORS-based Mouse Wound Healing Model: Histological Staining

**Fig. 6:**
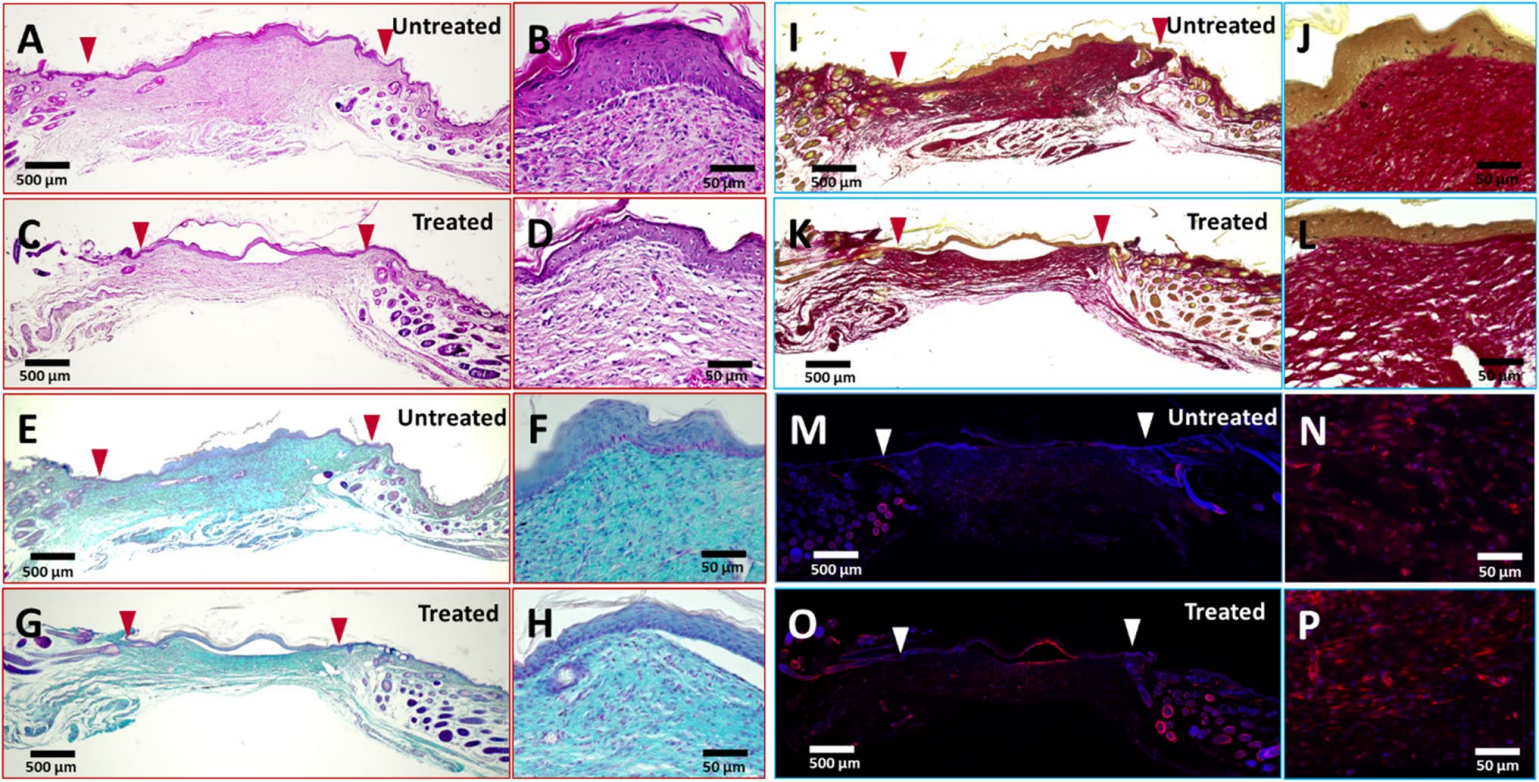
Histological staining and immunostaining on moMSCORS untreated and treated groups. Representative photos of histological cross-section of mouse wound bed with and without moMSCORS treatment at the final day 14. Whole range of histological cross-section of the wound skins are shown in (A, C, E, G, I, K, M, O), and higher magnification images of epidermis-dermis interface are shown in (B, D, F, H, J, L, N, P). Arrows demarcate the wound margins. (A, B) H&E staining in moMSCORS untreated group. (C, D) H&E staining in moMSCORS treated group. Nuclei are stained purple blue, and cytoplasm is stained pink. (E, F) Alcian Blue staining in moMSCORS untreated group. (G, H) Alcian Blue staining in moMSCORS treated group. Proteoglycan is stained blue, and nuclei are counterstained pink. (I, J) Picrosirius Red staining in moMSCORS untreated group. (K, L) Picrosirius Red staining in moMSCORS treated group. Collagen filaments are stained bright red, and nuclei are stained black. (M, N) αSMA immunostaining in moMSCORS untreated group. (O, P) αSMA immunostaining in moMSCORS treated group. Arrows demarcate the wound margins. αSMA is labeled with red fluorescence, and the nuclei are counterstained blue

On the final day 14 of the wound healing experiment, all the mice were sacrificed; the wound beds were histologically sectioned and stained in order to study the structural features of the wounds. Figure 6 shows representative histological staining by H&E, Alcian Blue and Picrosirius Red, as well as αSMA immunostaining of both treated and untreated groups.

H&E staining showed a well stratified and differentiated epithelium in all biopsies of the healed wounds. Remarkable difference in anatomical structure of the wound beds was noticed. The wound in the untreated group displayed a typical scarred hypertrophic tissue after wound healing, with a very thick epidermis (129.92±63.40 μm) and dermis (785.25±245.67 μm) (Figs. 5G and 6A). The alignment of the dermal collagen fibers was irregular and heterogeneous with the typical vertical orientation of blood vessels, with less vascularized and more inflammatory appearance (Fig. 6I, J). In contrast, the treated group displayed a distinguished wound bed structure, with 3-fold thinner epidermis (42.90±17.16 μm) and 2.2-fold dermis (359.08±137.05 μm), as shown in Figs. 5G and 6B. The thickness of both epidermis and dermis in the treated group was significantly lower than in the untreated group (***p=2.59 × 10^−5^ in epidermis, ***p=2.55 × 10^−6^ in dermis). The arrangement of dermal collagen filaments in the treated group was more regular and homogeneous, with an appearance closer to physiological (Fig. 6K, L) compared to the untreated group.

To investigate the dermal ECM production during the wound healing process, especially in terms of acid proteoglycan secreted by the dermal fibroblasts and myofibroblasts, Alcian Blue staining was employed for specimen staining of both treated and untreated groups (Fig. 6E-H). Alcian Blue staining of the mouse wound histological sections on day 14 showed abundantly stained proteoglycans in dermis in the wound bed of both groups. Consistent with the H&E staining, different wound healing outcome and ECM production was observed. In the skin of the untreated group, higher amount of proteoglycan was present, with irregularly, densely distributed collagen filaments. Intensive staining of Alcian blue indicated a higher deposition of proteoglycans in the untreated group with a stronger integration between epidermis and dermis as well as a higher amount of dermal cells and crosslinked dermal filaments. On the other hand, in the moMSCORS-treated group, the wounds displayed less intensive staining of Alcian Blue and a more uniform collagen fiber orientation within the dermis.

Picrosirius Red staining was employed to additionally visualize collagen fibers in the dermis. The observed fibrillary elements displayed a good contrast and clean texture, black-stained nuclei and dark brown epidermis. High amount of compact and fascicular collagen fibers were pronounced in the untreated wounds. moMSCORS-treated wounds showed a lower amount of thin horizontal, parallel collagen bundles, consistent with the outcome of the HE and Alcian Blue staining (Fig. 6I-L).

To investigate the presence and stage of the dermal fibroblasts/myofibroblast cycle at the final time point of day 14, αSMA immunostaining was used. αSMA immunostaining showed presence of distinguished dermal fibroblasts in dermis of both groups. Less αSMA-positive cells were observed in the hypertrophic scar of the untreated group than in the flattened wound bed of the moMSCORS-treated group (Fig. 6M-P). The number of cells, used to evaluate the cellularity of the wound bed, was higher in the wound bed of the untreated group (18.76±3.73 cells/1 × 10^4^ µm^2^) than that of the treated group (14.57±2.69 cells/1 × 10^4^ µm^2^, ***p=0.00087).

## Discussion

In this study, we established a platform of an immunocompetent grafting mouse model by isolating and culturing syngeneic mouse MSCs from the hair follicle ORS (moMSCORS) of the C57BL/6 mouse strain and by testing their safety and functionality in wound healing *in vivo*. Following an analog air-liquid migration-based isolation method for culturing of human MSCORS [32], moMSCORS were isolated, expanded *in vitro* and characterized according to the ISCT-defined Criteria for MSC classification [35]. The moMSCORS exhibited adherence to the cell culture plastic, a proliferative character, clear MSC expression profiles and capability of a multi-lineage differentiation. They expressed MSC markers CD44, CD73, CD90, CD105, and additionally Sca-1 and CD140a, nestin, CD117, CD133.

Except for the typical MSC biomarkers for MSC, additional markers Sca-1 and CD140a were used as a recently identified and selective combination for isolating mouse MSCs that are robust in both colony-forming and tri-lineage differentiation [36]. Sca-1, is the 18 kDa mouse glycosyl phosphatidylinositol-anchored cell surface protein, and it is widely used as the surface marker of mouse MSC [37]. CD140a, also known as the PDGFRα (Platelet-Derived Growth Factor Receptor Alpha), is regarded as an MSC marker [38]. Adherent, proliferative moMSCORS were capable of multi-lineage differentiation that were cultivated in this study also expressed Sca-1 and CD140a (Fig. 2A), in accordance with the reported landmarks.

The isolation process of moMSCORS from whisker hairs of the C57BL/6 mice differs with the MSCORS method due to the encapsulation of the mouse whisker hair follicle within a compact follicle-sinus complex. This complex comprises of horizontal spirals of tenacious and densely distributed fiber filaments, which support and stabilize the whisker hair follicles. Therefore, plucking of whisker hair follicles in order to isolate stem cells would result in losing a major portion of the ORS, which would remain in the tightly structured follicle-sinus complex. Micro-dissection, which was reported earlier [39, 40], has been used as an optimization step towards isolating intact ORS from the follicle-sinus complexes. This helps omitting prolonged enzymatic digestion of the ORS tissue and ensures that the ORS segment remains intact, attached and plucked along with the hair shaft. The combination of enzymatic digestion and microdissection granted not only an intact ORS, but also an exclusive, follicle ORS-related origin of the moMSCORS. This increased the isolation specificity and helped improve the efficiency of the previously reported methods, which frequently resulted in isolating stem cells from the residual dermal tissue attached to the follicle-sinus complex [39].

The wounds in the untreated control group were completely closed after 14 days, having developed hypertrophic epidermis and dermis, past the initial three stages of wound healing as previously reported [41]. The histological sectioning and staining enabled a finer grain for an assessment of the tissue structure and the thickness of the hypertrophic scarring (Fig. 6). The assessment showed: thicker epidermis and dermis, high number of fibroblasts in the dermis, high integration of the marginal tissue into the healed lesions, surplus of dermal matrix and compact, randomly oriented collagen fibers. All the observed parameters in the untreated group are in agreement with the expected over-healing effect that leads to forming of the hypertrophic scar tissue in wound healing under mechanical stimulation (Fig. 6A-D) [42]. In this study, the mechanical stimulation was caused by the mouse activity and a lack of dressing.

Contrary to the untreated wounds, all the mentioned parameters were reduced in the moMSCORS-treated wounds and the orientation of the collagen fibers was parallel and dense along with the reduced inflammation. This supports the conclusions from the existing reports using MSC application to wounds and monitoring their cell fate in mice [43]. Whereas the orientation of collagen fibers was consistent to that of a scar tissue, the moMSCORS-treated wounds were not hyper-cellularized and the scars were not hypertrophic, which all point out towards a more physiological wound healing outcome and in accord with the previously reported effect of adipose-derived MSCs in a mouse wound healing model [44].

Such outcome can be interpreted by a known immunomodulatory capacity of MSCs exerted by the means of employing cells with stem cell-like trophic properties, such as dermal fibroblasts, to negatively modulate inflammation related to non-healing wounds [5]. Thus, we aimed to look into the influence of moMSCORS on inflammation, i.e. whether their application changed the observed inflammatory parameters in terms of moderating edema, epithelization, cellularity, ECM deposition, angiogenesis, hypertrophy of the scar and the wound closure. After the moMSCORS were evenly distributed across the wound, those treated wounds exhibited a lower extent of inflammation than the untreated wounds according to the scored and quantified parameters of inflammation. As expected in the moMSCORS-treated wounds, re-epithelization and cellularity of the wound bed were less abundant, angiogenesis remained at a comparable level, wound closure occurred significantly faster, thickness of the scar tissue was significantly reduced and collagen fiber orientation was more physiological, on one side parallel and typical to that of the scar but with density closer to that of the native skin or non-hypertrophic scar (Fig. 5A, B, E-H, Fig. 6).

To the best of our understanding, absence of fibrosis and hypertrophic scarring can be explained by a previously reported paracrine, antifibrotic and anti-inflammatory effect of MSCs, known in wound healing [16, 45]. As previously reported, upon receiving stimulation by wound healing signals dermal fibroblasts undergo a transformation into myofibroblasts at early-to-intermediate stage [46]. In the present study, external application of moMSCORS suppressed local inflammation and differentiation of monocytes, hereby in all probability slowing down the activation of myofibroblasts, which would in the end lead to delaying the inflammatory phase. This might also be the reason that less dermal fibroblasts, dermal collagen filaments and a less extensive scar formation were found in the moMSCORS-treated group. In an attempt to look into the reduction on myofibroblast differentiation after moMSCORS treatment, we labelled the histological sections of wound beds with αSMA, a marker for myofibroblasts. At this point, the αSMA^+^ cells were more frequently present in the treated skin specimens, indicating that the untreated wound was past its intermediate phase (Fig. 6O). Whereas in the normal untreated skin, myofibroblasts differentiated to a large extent and vascular cells formed hypercellularized, collagen-rich scar tissue, pointing out to a later phase of wound healing (Fig. 6M).

On the other hand, the physiological capacity of wound healing in wild type mice may occlude the readout of the treatment effects and thus require employing a delayed wound model established in the diabetic db/db mice. Relevant *in vivo* studies reported by Franz et al. were carried out by applying an intradermal injection of activated human fibroblasts into the margin of a full-thickness dorsal excisional wound inflicted on db/db mice [47]. ln the resulting delayed wound healing context, typical for db/db mice, fibroblasts reduced the inflammation as well as increased the cellularity and the tissue volume. The method of full-thickness dorsal wounds and the analytical criteria for inflammation in our study strongly diverged from the procedures used in the aforementioned study [47]. We cultured murine moMSCORS in order to by-pass possible occlusions caused by xenogeneic transplantation. By establishing a syngeneic context of donors and recipients, both immunocompetent and simultaneously autologous grafting mouse model was established, addressing the question of modifications in physiological wound healing caused by moMSCORS rather than that of the delayed wound healing. The main stages of physiological wound healing in the immune- and wound healing-competent C57BL/6 animals inherently occurred both with and without the moMSCORS grafting, thus the differences in inflammation parameters were not as prominent as in the delayed wound healing models. Nevertheless, the intensive inflammation in both treated- and untreated animals did not occlude the differences in studied inflammation parameters between groups, as quantified by the scoring system used in this study. This confirms the sufficient sensitivity of the readout and suitability of the applied experimental mouse model.

As a murine equivalent to human MSC from hair follicle ORS (MSCORS), moMSCORS presented a base for a homolog, syngeneic mouse model involving mouse MSCs from hair follicle, which are developmentally, phenotypically and functionally comparable to the human MSCORS. The developmental potential of human MSCORS has already been previously highlighted [32]. The interaction between the human MSCORS and immune-challenged mouse host would completely bypass the physiological immune response. The selected homologous syngeneic model in immunocompetent animals is recommended by EMA [48], and it is clearly suitable for moMSCORS, as an equivalent of autologous grafting. In this study, the syngeneic moMSCORS cell line provided an elegant and accurate approach in an immunocompetent C57BL/6 context and it delivers an output identical to that of an autologous application, in scientific as well as in translational regulatory terms.

The model applied brought about certain further limitations too. Apparently, in the untreated group the thickness of fibrin crust and scab was higher than in the treated group, it was consequently discharged earlier and more aptly during the maturation stage. The thinner scab in the moMSCORS-treated group persisted for a longer time. For those reasons, which are inherent to the applied wound-healing-competent model, we found it necessary to calibrate the analysis of inflammation parameters by assessing only non-occluded wounds with a clear visual access to the wound. Further *in vivo* research is required to investigate the impacts of moMSCORS on the wound bed, including examining the production of the pro-inflammatory factors, survival time of grafted moMSCORS and the time window of fibroblast/myofibroblast differentiation.

In summary, this study established a culture of syngeneic mouse MSC (moMSCORS) from the whisker hair follicle of a mouse as a cellular agent for a syngeneic, immunocompetent *in vivo* mouse model of wound healing. The moMSCORS-treated wounds presented moderating effects, exhibited in acceleration of the wound closure, reduction of the cellularity, dermal ECM deposition, myofibroblast phenotype, scar formation and size, suggesting changes in the inflammation and proliferation phase. Those effects may proof helpful in treating non-healing wounds and preventing hypertrophic scars in future.

## Data Availability

The data generated during the current study are available from the corresponding author on reasonable request.
